# Western dietary pattern during pregnancy and early childhood increases risk of childhood asthma dependent on socioeconomic status

**DOI:** 10.1111/pai.70351

**Published:** 2026-04-17

**Authors:** Mina Ali, David Horner, Tingting Wang, Nicklas Brustad, Min Kim, Liang Chen, Ann‐Marie Schoos, Nilo Vahman, Augusto Litonjua, Scott T. Weiss, Jessica Lasky‐Su, Klaus Bønnelykke, Jakob Stokholm, Morten A. Rasmussen, Bo L. Chawes

**Affiliations:** ^1^ COPSAC, Copenhagen Prospective Studies on Asthma in Childhood, Herlev and Gentofte Hospital University of Copenhagen Copenhagen Denmark; ^2^ Department of Pediatrics Slagelse Hospital Slagelse Denmark; ^3^ Department of Clinical Medicine, Faculty of Health and Medical Sciences University of Copenhagen Copenhagen Denmark; ^4^ Division of Pediatric Pulmonary Medicine, Golisano Children's Hospital University of Rochester Medical Center Rochester New York USA; ^5^ Channing Division of Network Medicine Brigham and Women's Hospital, Harvard Medical School Boston Massachusetts USA; ^6^ Section of Microbiology and Fermentation, Dept of Food Science University of Copenhagen Copenhagen Denmark

**Keywords:** childhood asthma, metabolomics, socioeconomic status, Western dietary pattern

## Abstract

**Background:**

A Western dietary pattern (WDP) is linked to socioeconomic status (SES) and has been proposed as a risk factor for childhood asthma. However, how SES influences the relationship between dietary patterns and asthma risk remains poorly understood.

**Objective:**

We aimed to investigate the interplay between SES and WDP during pregnancy and early childhood on risk of childhood asthma.

**Methods:**

We analyzed 594 mother–child pairs from the COPSAC_2010_ cohort. Maternal food frequency questionnaires (FFQs) were completed during pregnancy, and children were followed for asthma/wheeze until age 10 years. Untargeted plasma metabolomics profiles were obtained at pregnancy week 24 and child ages 6 and 18 months. A WDP score derived from FFQs was used to compute WDP metabolite scores using sparse partial least squares (sPLS). Associations between WDP and asthma/wheeze and their interactions with SES were evaluated by regression models. Replication was performed in 772 mother–child pairs from VDAART with comparable metabolomics data and in 388 children from COPSAC_2000_ with neonatal dried blood spot metabolomics. SES was defined using principal component analysis of household income, maternal education, and maternal age.

**Results:**

In COPSAC_2010_, SES was inversely correlated with WDP (*r* = −0.40 to −0.14; *p* < .01). Significant WDP–SES interactions were observed for multiple asthma/wheeze outcomes (*p* < .05). Among children with low SES, higher maternal WDP FFQ‐scores were associated with an increased risk of recurrent wheeze until 3 years (aHR = 1.29 [1.03–1.60], *p* = .023) and asthma until 10 years (aHR = 1.32 [1.06–1.65], *p* = .015) and higher child WDP metabolite‐score were associated with asthma until 10 years (aHR = 1.87 [1.21–2.90], *p* = .005). No significant WDP–SES interactions were observed in the replication cohorts; however, low SES was independently associated with asthma/wheeze in VDAART, and higher WDP‐scores were associated with increased risk in COPSAC_2000_ among low‐SES children.

**Conclusions:**

WDP during pregnancy and early childhood is associated with increased risk of childhood asthma/wheeze, with evidence supporting a directional association with SES rather than effect modification.

**Abbreviations**
BMI: Body mass index
CMPF: 3‐carboxy‐4‐methyl‐5‐propyl‐2‐furan propanoic acid
COPSAC_2000_
: Copenhagen Prospective Studies on Asthma in Childhood 2000
COPSAC_2010_
: Copenhagen Prospective Studies on Asthma in Childhood 2010
FFQ: Food frequency questionnaire
IU: International unit
LC–MS: Liquid chromatography mass spectrometry
n‐3 LCPUFA: n‐3 long chain polyunsaturated fatty acids
PCA: Principal component analysis
PRS: Polygenic risk score
RCT: Randomized controlled trial
SES: Socioeconomic status
SNP: Single‐nucleotide polymorphism
sPLS: Sparse partial least square
VDAART: Vitamin D Antenatal Asthma Reduction Trial
WDP: Western dietary pattern

**Key message**

A Western dietary pattern and low socioeconomic status have been linked to childhood asthma, but their interplay remains poorly understood. In this Danish mother–child cohort study, combining FFQ data with longitudinal metabolome profiles, we found that higher maternal and child Western diet scores were associated with an increased risk of asthma and wheeze, particularly among children from families with lower socioeconomic status. In replication analyses in two independent cohorts from Denmark and the United States, we found evidence supporting directional associations with SES, but not an interaction effect. This suggests that dietary screening and targeted interventions during pregnancy may help prevent childhood asthma.

## 1 INTRODUCTION

Asthma is a chronic airway disorder with a complex interplay of genetic, environmental, and behavioral factors.^1^, ^2^ Shifts in diet, physical inactivity, and environmental exposures associated with industrialized lifestyles have been linked to the rising prevalence of asthma, although the exact mechanisms remain unclear.^3^, ^4^ A Western dietary pattern (WDP), characterized by higher intakes of refined grains, red meat, processed food, and sugar, along with relatively lower intake of fruits, vegetables, and whole grains, is considered a risk factor for asthma.^5^, ^6^ This type of diet is associated with obesity and chronic inflammation, which affect asthma risk and severity.^3^, ^7^ The association of a WDP with the risk of asthma may be particularly prominent during pregnancy, as the diet in pregnancy has a fetal programming effect that can alter lung development and increase the risk of asthma in childhood.^8^ Poor maternal diet may impair fetal lung development through nutrient deficiencies,^8^, ^9^ inflammation,^10^ oxidative stress,^11^, ^12^ and epigenetic changes.^13^

Despite observations suggesting a connection between WDP and increasing asthma prevalence,^3^, ^4^, ^5^ conclusive evidence and clinical studies directly linking dietary patterns with asthma are still lacking. A growing body of evidence also emphasizes the critical role of socioeconomic status (SES) in the increasing prevalence, development and management of asthma, where lower SES is associated with higher disease burden, highlighting the uneven social distribution of asthma.^14^, ^15^, ^16^ Together, these findings suggest that both nutritional patterns during pregnancy and early childhood, and the SES in which children grow and develop, play a critical moderating role in the development of asthma. Many studies have shown associations between low SES and unhealthy dietary patterns, such as the WDP;^17^, ^18^, ^19^ however, whether and how SES influences the relationship between dietary patterns and asthma risk remains poorly investigated.

In this study, we hypothesized that both WDP and SES are involved in the development of childhood asthma and used an exploratory analytical approach to investigate their interaction in relation to asthma outcomes, hypothesizing SES as an effect modifier. We explored this complex relationship within the Copenhagen Prospective Studies on Asthma in Childhood 2010 (COPSAC_2010_) population‐based cohort of 700 mother–child pairs and replicated the findings in two independent cohorts: the COPSAC_2000_ birth cohort, which includes 388 children of asthmatic mothers, and the US‐based Vitamin D Antenatal Asthma Reduction Trial (VDAART) birth cohort, comprising 876 pregnant women with either a history of asthma or allergies in themselves or the biological father and their 810 offspring.

In a previously published study,^20^ we employed a methodological framework that combined food frequency questionnaires (FFQ) with longitudinal untargeted plasma metabolomics profiling to investigate the impact of maternal diet during pregnancy on the risk of neurodevelopmental disorders in children from the COPSAC_2010_ cohort, and we replicated the findings in three other cohorts, including COPSAC_2000_ and VDAART. In the present study, we used the same indexes to assess the WDP during pregnancy and early childhood. For SES, we considered household income, maternal education level, and maternal age at the child's second birthday. To our knowledge, this is the first clinical study to investigate the interplay between WDP and its metabolite composition, SES factors, and childhood asthma outcomes.

**FIGURE 1:**
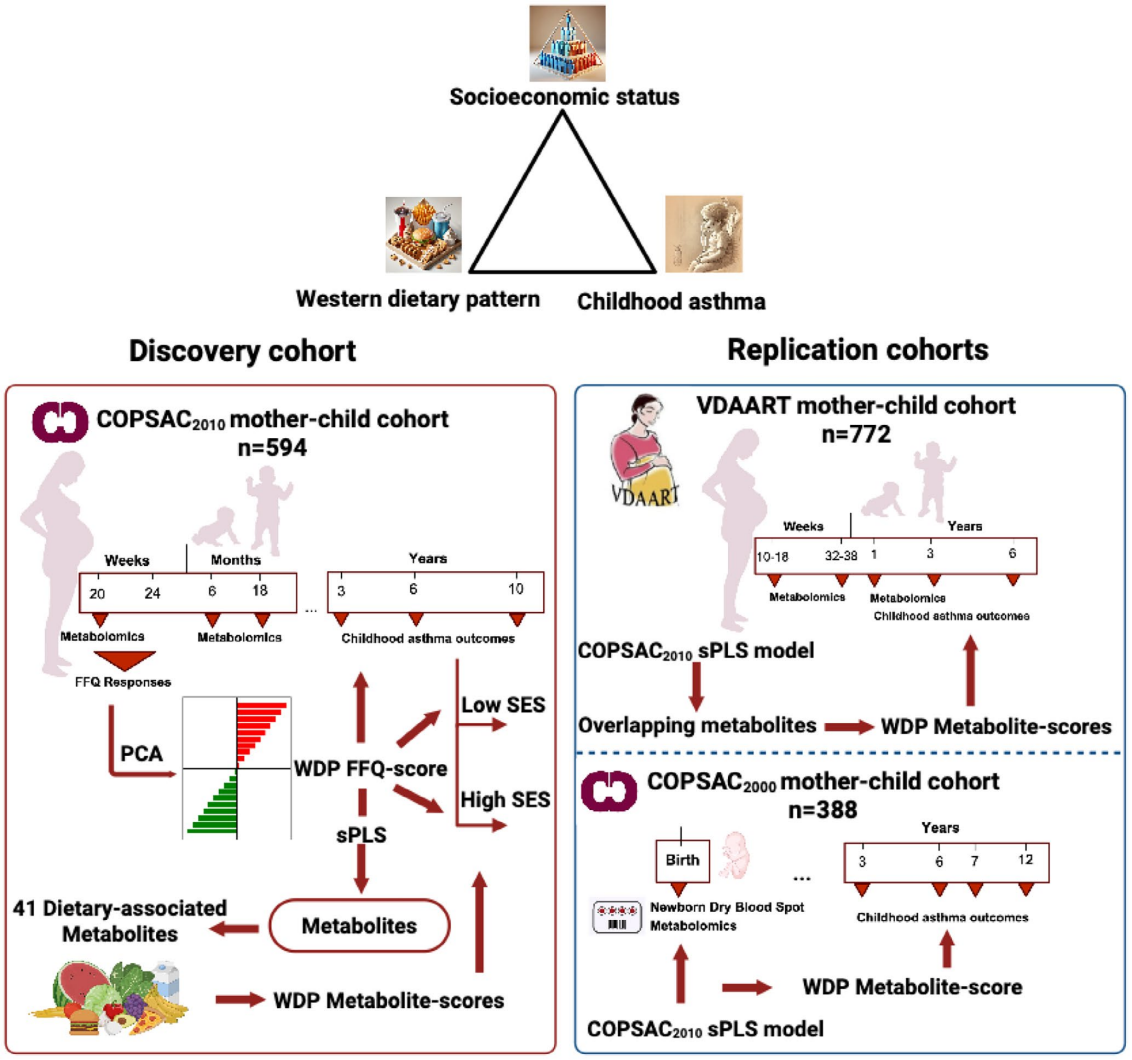
Overview of the study design. The methodological framework outlines the investigation of the interplay between the Western dietary pattern (WDP), socioeconomic status (SES), and childhood asthma. WDP was represented by the second principal component (PC) derived from FFQ responses, which was used to identify WDP‐related metabolites and compute WDP metabolome scores. SES was derived from the first principal component of a PCA that included household income, maternal education level, and maternal age at childbirth.

An overview of the study design is shown in Figure 1.

## 2 METHODS

### 2.1 Study design and participants

The COPSAC_2010_ is a Danish population‐based cohort of 700 mother–child pairs designed to study early‐life factors influencing asthma development.^21^ It involves extensive clinical longitudinal phenotyping through 14 visits from birth until age 10 years. In this study, we investigated 594 mother–child pairs from the COPSAC_2010_ cohort who completed FFQs between weeks 20 and 24 of pregnancy with realistic energy intake values (between 4200 kJ/day and 16,700 kJ/day).

In replication analysis, we utilized two independent cohorts: the US‐based VDAART cohort^22^ and the COPSAC_2000_ cohort.^23^ In VDAART, 876 pregnant women with a history of asthma or allergies in themselves or in the biological father were enrolled between 10 and 18 weeks of gestation. The children attended yearly scheduled visits until age 6. COPSAC_2000_ included 388 children born to asthmatic mothers from 1999 to 2001. The children attended scheduled clinical visits every 6 months until age 7 years, 12 and 18 years and whenever acute respiratory, allergy or skin symptoms manifested.

The COPSAC_2000_ and COPSAC_2010_ studies are conducted according to the principles of the Declaration of Helsinki and are approved by the Ethics Committee of Copenhagen (KF 01–289/96, H‐B‐2008‐093) and the Danish Data Protection Agency (2015‐41‐3696). Both parents gave oral and written informed consent before enrolment.

The VDAART obtained written informed consent from all pregnant participants. The study was approved by the Institutional Review Boards at all participating centers and the Data Coordinating Center, with oversight from a Data Safety Monitoring Board.

### 2.2 Metabolome profiling and data preprocessing

Untargeted plasma metabolome profiling from COPSAC_2010_ (mothers at pregnancy week 24 and children at ages 6 and 18 months) and VDAART (mothers at pregnancy weeks 10–18 and 32–36 and children at ages 1 and 3 years) was carried out by Metabolon, Inc. (North Carolina, USA) as previously detailed.^24^ For COPSAC_2010_, twenty‐eight anchor samples were included at all time points and were used for the calculation of scaling factors and the adjustment of the batch‐normalized data at different time points. Metabolites with more than 30% missing data were removed from the analysis, and the remaining missing data were imputed with the minimum value for each metabolite. The metabolome data were log‐transformed before analysis. For VDAART, the metabolome data were log‐transformed, centered, and scaled prior to analysis.

For COPSAC_2000_, the metabolome profile was acquired from neonatal dried blood spots (DBS) using liquid chromatography–mass spectrometry (LC–MS). The DBS samples were collected at ages 1–12 days after birth and were stored at −20°C at the Danish National Biobank until analysis. A detailed description of the sample preparation, LC–MS metabolic profiling, and data preprocessing can be found in previous work.^25^

### 2.3 Western dietary pattern derived from FFQ

In COPSAC_2010_, the FFQ consisted of 360 items of food and beverages, grouped into 95 nutrient constituents, as detailed in our previously published study.^20^ From this study, we used the second principal component (PC2) from a principal component analysis (PCA) of the 95 nutrient constituents based on FFQ responses collected during pregnancy, after excluding mothers who reported unrealistic energy intakes below 4200 kJ d^−1^ or above 16,700 kJ d^−1^, as an index of a WDP (Figure S1A). In this analysis, PC1 explained 44.3% of the total variance and was positively associated with all food groups, representing a “Varied dietary pattern”. PC2 explained 10.7% of the variance and was positively associated with intakes of animal fats, refined grains, and high‐energy drinks, while being negatively associated with intakes of fruit, fish, and vegetables, thus representing a WDP, which is subsequently referred to as the WDP FFQ‐score.

### 2.4 Western dietary pattern derived from metabolome profiles

In COPSAC_2010_, the WDP FFQ‐score was utilized as the response variable to identify WDP‐related metabolites using a sparse partial least squares (sPLS) regression model with cross‐validated predictions (repeated cross‐validation with 5 folds and 10 repeats), implemented in the caret package (v6.0.90)^26^ in R. In the best blood metabolome model for predicting the pregnancy WDP, 41 metabolites survived regularization in the sPLS (RMSECV = 0.87, *R* ^2^CV = 0.24). The loadings of these metabolites are shown in Figure S1B. WDP metabolite‐scores were predicted based on the weights of these metabolites for pregnancy week 24 (586 samples) and child ages 6 (508 samples) and 18 months (515 samples). In all analyses, we considered the WDP FFQ‐score and the derived WDP metabolite‐scores as WDP exposure measures.

In VDAART, which included metabolome profiling of mothers at early (weeks 10–18) and late (weeks 32–38) pregnancy, as well as child ages 1 and 3 years, we applied COPSAC_2010_ cohort‐trained models to calculate WDP metabolite‐scores at all time points using overlapping metabolites (640 in early pregnancy, 689 in late pregnancy, 523 at 1 year, and 530 at 3 years). The predicted scores were subsequently scaled, and thus results are interpreted as per SD change in the given population.

In COPSAC_2000_, the same approach was applied to calculate WDP metabolite‐scores using the DBS metabolome data collected at age 1–12 days. There were initially 1253 overlapping metabolites (both named and unnamed) identified between the COPSAC_2010_ and COPSAC_2000_ cohorts. After filtering for metabolites with a cross‐correlation of <0.4 between cohorts, 951 overlapping features remained for analysis.

### 2.5 Socioeconomic status

In all cohorts, the SES variable was determined using PC1 from a PCA that included household income, maternal education level, and maternal age at the child's second birthday. SES components were assessed at the child's second birthday rather than at delivery to reduce potential misclassification, as income and educational attainment may change in the years following pregnancy, particularly among mothers still completing their education. To avoid misclassifying these mothers as low SES, we hypothesized that the risk of baseline misclassification would be greater than the potential for postexposure misclassification.

In the COPSAC cohorts, annual household income was categorized into five groups: <50,000 EUR, 50,000–80,000 EUR, 80,000–110,000 EUR, 110,000–135,000 EUR, and >135,000 EUR. Maternal education was categorized into three levels: college or less, medium‐length academic education or tradesman's certification, and university degree or higher. In COPSAC_2010_, PC1 accounted for 55% of the total variance (loadings: income = 0.62, education = 0.57, and age = 0.53), while in COPSAC_2000_, PC1 captured 52% of the total variance (loadings: income = 0.59, education = 0.54, and age = 0.60).

In the VDAART cohort, household income was categorized into six groups: <30,000 USD, 30,000–50,000 USD, 50,000–75,000 USD, 75,000–100,000 USD, 100,000–150,000 USD, and >150,000 USD. Maternal education was classified into four levels: Did not graduate from high school, Graduated from high school/technical school, Junior college/some college, and Graduate school/College graduate. In VDAART, PC1 accounted for 72% of the total variance (loadings: income = 0.60, education = 0.58, and age = 0.54).

### 2.6 Clinical asthma/wheeze outcomes

In the COPSAC cohorts, clinical asthma and wheeze outcomes included the number of episodes with asthma‐like symptoms up to age 3, recurrent wheeze up to age 3, the number of asthma/wheeze exacerbations up to age 6, and asthma diagnosis up to age 6 and 10 years.

Asthma‐like symptoms were defined by 3 consecutive days with significant cough, wheeze, or dyspnea and were recorded by the parents in a day‐to‐day diary chart as a dichotomized daily score (yes/no) from birth until age 6–7 years.^27^, ^28^

Recurrent wheeze was defined by 5 episodes of asthma‐like symptoms each lasting at least 3 consecutive days within 6 months or asthma‐like symptoms for at least 4 consecutive weeks.

Asthma diagnosis was based on recurrent wheeze, typical asthma symptoms such as prolonged nocturnal cough and recurrent cough outside common cold, need for short‐acting inhaled beta‐2‐agonist, and response to a 3‐month trial of inhaled corticosteroids (ICS) with relapse upon cessation.^28^

Asthma exacerbations at age 0–6 years were defined as symptoms requiring acute care visits or hospitalization, or oral or high‐dose ICS treatment in addition to daily ICS treatment.

In VDAART, the diagnosis of asthma was based on parental reports of physician‐diagnosed asthma and parental report of recurrent wheeze until ages 3 and 6 years.^22^ The physician's diagnosis of asthma was based on a history of recurrent wheezing episodes, use of asthma medications, and clinical symptoms such as persistent cough and difficulty breathing.

The asthma and wheeze outcomes in the COPSAC and VDAART cohorts are summarized in Table S9.

### 2.7 Covariates

In the multivariable analyses, the following covariates were included: prepregnancy maternal body mass index (BMI), gestational age, cesarean delivery, smoking during pregnancy, alcohol consumption during pregnancy, child sex, birth weight, birth season, duration of exclusive breastfeeding, daycare start age, SES, and prenatal n‐3 long chain polyunsaturated fatty acids (n‐3 LCPUFA)^29^ and high‐dose vitamin D interventions.^30^

To study the genetic impact on the associations between WDP and asthma/wheeze outcomes, we included a polygenic risk score (PRS) for childhood asthma in the COPSAC cohorts. Child genotyping was performed in children with Caucasian ethnicity using the Illumina Infinium HumanOmniExpressExome BeadChip. Single‐nucleotide polymorphisms (SNPs) for the PRS were derived from a genome‐wide association study (GWAS) on 142,000 individuals of diverse ancestry.^31^ The PRS‐continuous shrinkage (cs) method^32^ was used to construct the score using the 1000 Genomes reference panel. The final score consisted of 985,852 SNPs available in the GWAS summary statistics, reference panel, and the COPSAC_2010_ cohort.

### 2.8 Computational and statistical analysis

Associations between maternal WDP FFQ‐score and maternal and child WDP metabolite‐scores with asthma/wheeze outcomes were investigated using Cox and quasi‐Poisson regression for longitudinal data, and logistic regression for cross‐sectional data in the cohorts. The proportional hazards assumption in Cox models was assessed using Schoenfeld residuals (cox.zph in R) to evaluate whether the effects of WDP scores on asthma outcomes were constant over time. No evidence of nonproportionality was observed for WDP FFQ or metabolite scores in any of the analyses. To examine the interaction effect of WDP with SES and PRS in relation to asthma/wheeze outcomes, we added cross‐products to the models. All multivariable models were adjusted for the previously mentioned covariates.

PCA and forest plot visualizations were generated using the R package ggplot2. A significance level of 0.05 was used in all analyses, and to account for multiple testing, false discovery rate (FDR) control at 5% was applied using the Benjamini–Hochberg procedure within each outcome across the four WDP‐related scores and for the interaction analyses. All analyses were done in R version 4.3.2.

## 3 RESULTS

### 3.1 Baseline characteristics

This study included 594 mother–child pairs from the COPSAC_2010_ cohort who completed the FFQ between weeks 20 and 24 of pregnancy. Baseline characteristics and clinical measures of the mothers and their children, for subgroups with and without FFQ responses, are outlined in Table S1. The only significant difference observed was related to the birth season, with the subgroup without FFQ responses having a higher number of children born in winter and a lower number born in other seasons.

Replication was sought in the VDAART and COPSAC_2000_ cohorts, and Table 1 shows a comparison of baseline characteristics between the included participants from the COPSAC_2010_ (*n* = 594), VDAART (*n* = 772), and COPSAC_2000_ (*n* = 388) cohorts. COPSAC_2010_ showed significant differences compared with the other cohorts in many characteristics, particularly higher maternal age at childbirth, maternal education level, and annual income, indicating a higher SES.

**TABLE 1:** Baseline characteristics of mothers and children in the COPSAC_2010_, VDAART and COPSAC_2000_ cohorts.

Baseline characteristics	COPSAC_2010_	VDAART	COPSAC_2000_
**Mothers**			
Number	594	772	388
Age at birth (years) (mean [SD])*+	32.2 (4.3)	27.4 (5.5)	30 (4.5)
Gestational age at birth (days) (mean [SD])*+	279.2 (11.3)	273.9 (11.4)	280.6 (10.0)
Maternal prepregnancy Body Mass Index (mean [SD])*	24.5 (4.3)	28.3 (7.7)	Na
Smoking during pregnancy (Yes/No)*+	48/546	18/754	95/293
Maternal education at birth (Low/Medium/High)*+	43/380/171	327/183/262	205/100/54
Annual income (Low/Medium/High)*+	52/319/223	227/196/86	112/217/30
**Children**			
Number	594	459	388
Gender, Female/ Male	286/308	214/245	197/191
Caucasian Race (%)*	571 (96.1)	150 (32.7)	374 (96.4)
Birth weight (Kg) (mean (SD))*	3.56 (0.5)	3.3 (0.5)	3.51 (0.5)
Birth season Winter/Spring/Summer/Fall	143/177/139/133	112/113/105/129	72/88/91/96
Cesarean delivery (Yes/No)*	130/464	119/289	78/310
Asthma ever at age 10 (Yes/No)+	154/440	118/323	44/258

*Note*: In the COPSAC cohorts, low, medium, and high educational attainment are defined as “primary, secondary, or college graduate,” “tradesman or bachelor's degree,” and “master's degree or higher,” respectively. In VDAART, these levels are defined as “Did not graduate from high school/Graduated from high school,” “Technical school/Junior college/some college,” and “Graduate school/College graduate”. Low, medium, and high income are defined as <50,000, 50,000–110,000, and >110,000 euros in the COPSAC cohorts, and <$30,000, $30,000–$99,999, and >$100,000 USD in VDAART. * indicates a significant difference (*p* < .05) between COPSAC_2010_ and VDAART, and + indicates a significant difference (*p* < .05) between COPSAC_2010_ and COPSAC_2000_.

Abbreviation: Na, not available.

### 3.2 Association between WDP and asthma/wheeze outcomes in COPSAC_2010_

In multivariable models adjusted for covariates, a higher WDP metabolite‐score at pregnancy week 24 was associated with more asthma‐like symptoms until age 3 (aIRR = 1.07 [95% CI, 1.02–1.12], *p* = .007, FDR‐adjusted *p* = .028) (Table S2). No other significant associations were observed between WDP scores and childhood asthma outcomes.

### 3.3 Interplay between WDP, SES, and childhood asthma/wheeze in COPSAC_2010_

We observed strong negative correlations between SES and WDP scores during pregnancy and early childhood (Pearson correlation coefficients ranging from −0.40 to −0.14, FDR‐corrected *p*‐values<0.01) (Figure S2A). Thereafter, we investigated the possible interaction between WDP during pregnancy and early childhood and SES in relation to the risk of childhood asthma and wheeze outcomes. For pregnancy WDP, there was a significant interaction between maternal WDP FFQ‐score and SES for the risk of recurrent wheeze until age 3 years (*p*‐interaction = 0.019, FDR‐adjusted *p* = .064), asthma until age 6 (*p*‐interaction = 0.037, FDR‐adjusted *p* = .074), and 10 years (*p*‐interaction = 0.023, FDR‐adjusted *p* = .04) in multivariable models adjusted for covariates. For early childhood, a significant interaction was found between child WDP metabolite‐score at 6 months and SES for recurrent wheeze until age 3 (*p*‐interaction = 0.032, FDR‐adjusted *p* = .064), and asthma until age 10 years (*p*‐interaction = 0.030, FDR‐adjusted *p* = .039). Additionally, significant interactions were found between the child WDP metabolite‐score at 18 months and the number of asthma‐like symptoms until age 3 (*p*‐interaction = 0.004, FDR‐adjusted *p* = .016), and risk of asthma until age 6 (*p*‐interaction = 0.014, FDR‐adjusted *p* = .056) and 10 (*p*‐interaction = 0.008, FDR‐adjusted *p* = .032) (Table 2).

**TABLE 2:** Association of WDP scores with childhood‐asthma outcomes in low and high SES groups in the COPSAC_2010_ cohort in multivariate analysis.

	Analysis	SES	FFQ‐score pregnancy (week 24–28)	Interaction *p*‐value	Metabolite‐score pregnancy (week 24)	Interaction *p*‐value	Metabolite‐score child 6 months	Interaction *p*‐value	Metabolite‐score child 18 months	Interaction *p*‐value
Asthma‐like symptoms at age 0–3 years	Quasi‐Poisson	Low	1.00 [0.96–1.05] (*p* = .893) (*n* = 273)	0.412	1.04 [0.97–1.10] (*p* = .263) (*n* = 268)	0.294	1.00 [0.91–1.10] (*p* = .978) (*n* = 236)	0.655	0.91 [0.82–1.02] (*p* = .106) (*n* = 240)	0.004 (*q* = 0.016)
		High	1.06 [0.99–1.12] (*p* = .085) (*n* = 279)		1.08 [1.01–1.16] (*p* = .026, *q* = 0.104) (*n* = 276)		1.07 [0.95–1.20] (*p* = .268) (*n* = 238)		1.06 [0.93–1.20] (*p* = .367) (*n* = 249)	
Recurrent wheeze at age 0–3 years	Cox	Low	1.29 [1.03–1.60] (*p* = .023, *q* = 0.092) (*n* = 290)	0.019 (*q* = 0.064)	1.09 [0.85–1.38] (*p* = .497) (*n* = 285)	0.328	1.41 [0.95–2.11] (*p* = .089) (*n* = 251)	0.032 (*q* = 0.064)	1.19 [0.76–1.87] (*p* = .453) (*n* = 254)	0.355
		High	0.95 [0.74–1.24] (*p* = .726) (*n* = 286)		0.93 [0.71–1.22] (*p* = .604) (*n* = 283)		0.68 [0.44–1.05] (*p* = .082) (*n* = 242)		1.39 [0.85–2.27] (*p* = .193) (*n* = 255)	
Number of exacerbations at age 0–6 years	Quasi‐Poisson	Low	1.22 [0.94–1.56] (*p* = .124) (*n* = 290)	0.138	1.27 [0.95–1.70] (*p* = .110) (*n* = 285)	0.093	2.11 [1.27–3.59] (*p* = .005, *q* = 0.02) (*n* = 251)	0.094	1.28 [0.73–2.25] (*p* = .398) (*n* = 254)	0.442
		High	0.77 [0.52–1.13] (*p* = .197) (*n* = 286)		0.68 [0.46–1.00] (*p* = .055) (*n* = 283)		0.71 [0.39–1.30] (*p* = .265) (*n* = 242)		0.93 [0.46–1.87] (*p* = .848) (*n* = 255)	
Asthma at age 0–6 years	Cox	Low	1.24 [0.98–1.59] (*p* = .077) (*n* = 290)	0.037 (*q* = 0.074)	1.09 [0.83–1.43] (*p* = .546) (*n* = 285)	0.117	1.95 [1.21–3.13] (*p* = .006, *q* = 0.024) (*n* = 251)	0.068	1.41 [0.84–2.35] (*p* = .193) (*n* = 254)	0.014 (*q* = 0.056)
		High	0.80 [0.59–1.09] (*p* = .160) (*n* = 286)		0.84 [0.61–1.14] (*p* = .252) (*n* = 283)		0.80 [0.49–1.32] (*p* = .392) (*n* = 242)		0.67 [0.38–1.17] (*p* = .161) (*n* = 255)	
Asthma at age 0–10 years	Cox	Low	1.32 [1.06–1.65] (*p* = .015, *q* = 0.03) (*n* = 290)	0.023 (*q* = 0.040)	1.13 [0.87–1.45] (*p* = .357) (*n* = 285)	0.231	1.87 [1.21–2.90] (*p* = .005, *q* = 0.02) (*n* = 251)	0.030 (*q* = 0.039)	1.51 [0.94–2.44] (*p* = .091) (*n* = 254)	0.008 (*q* = 0.032)
		High	0.80 [0.61–1.05] (*p* = .108) (*n* = 286)		0.86 [0.66–1.13] (*p* = .290) (*n* = 283)		0.81 [0.52–1.26] (*p* = .345) (*n* = 242)		0.62 [0.38–1.03] (*p* = .067) (*n*= 255)	

*Note*: The analysis is adjusted for prepregnancy maternal BMI, gestational age, cesarean delivery, maternal smoking and alcohol consumption during pregnancy, child sex, birth weight, birth season, length of solely breastfeeding, daycare start age and n‐3 LCPUFA and vitamin D interventions. To measure SES, we included household income, maternal education level, and maternal age at the child's second birthday in a PCA model. Interaction *p*‐values correspond to the *p*‐values for the interaction between WDP scores and SES in multivariate analysis. *q* denotes the Benjamini‐Hochberg adjusted P value.

**FIGURE 2:**
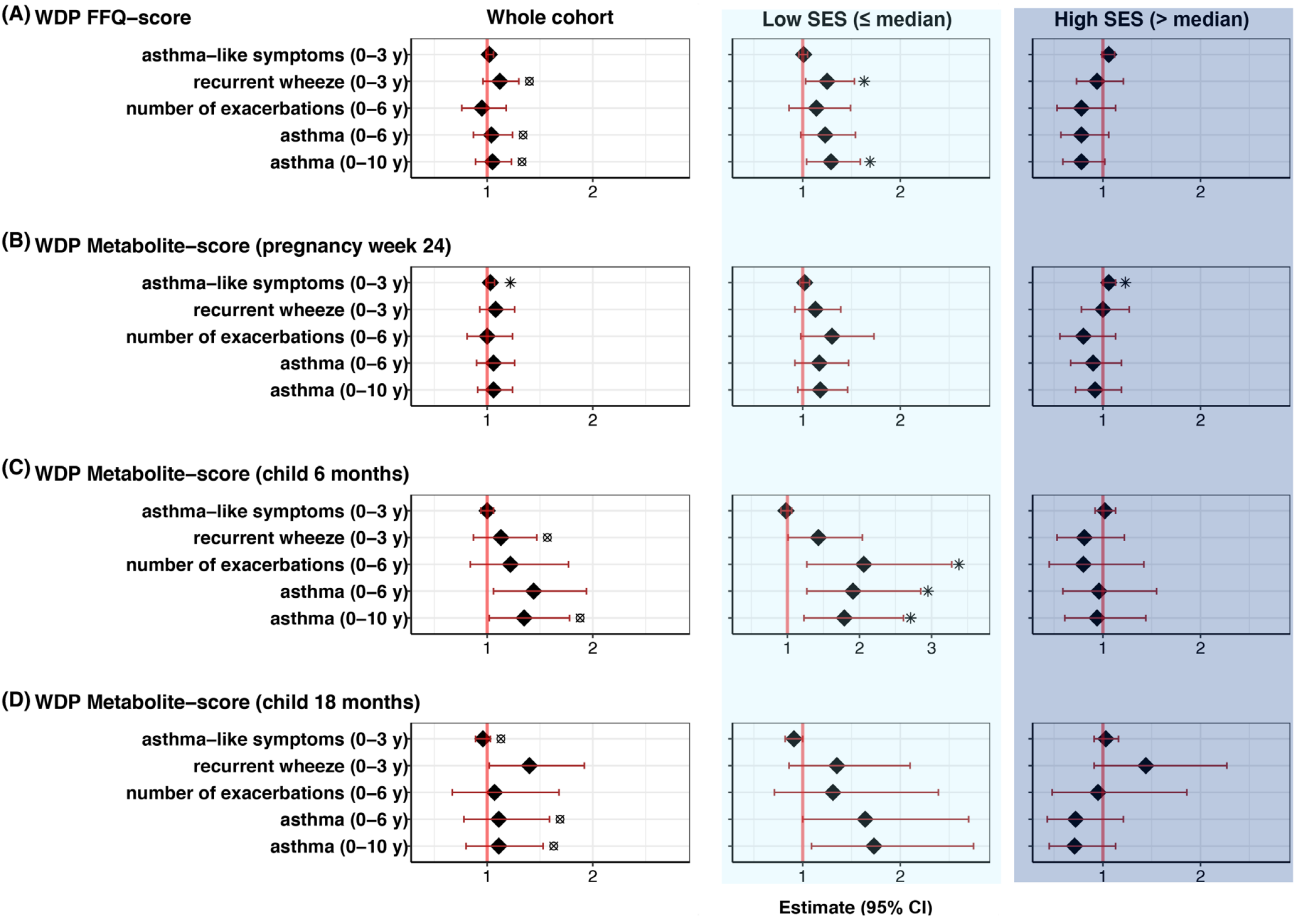
Association of WDP scores with childhood asthma/wheeze outcomes in the entire COPSAC_2010_ cohort and in subgroups stratified by median SES. The figures are based on univariate analyses. * Indicates a significant association in multivariate analysis, ⊠ indicates a significant interaction between SES and WDP score in multivariate analyses (*p* < .05).

In multivariable covariate adjusted models stratified into low and high SES by a median split, we observed a general pattern that higher WDP scores in pregnancy and early childhood were associated with an increased risk of asthma and wheeze outcomes among children with low SES, but not among children with high SES (Figure 2). For example, a higher maternal WDP FFQ‐score was associated with a higher risk of recurrent wheeze until age 3 and asthma until age 10 among children with low SES (aHR = 1.29 [1.03–1.60], *p* = .023 and aHR = 1.32 [1.06–1.65], *p* = .015, FDR‐adjusted *p* = .03), but not among children with high SES (aHR = 0.95 [0.74–1.24], *p* = .726 and aHR = 0.80 [0.61–1.05], *p* = .108). Similarly, a higher child WDP metabolite‐score at age 6 months was associated with a higher risk of asthma until age 10 among children with low SES (aHR = 1.87 [1.21–2.90], *p* = .005, FDR‐adjusted *p* = .02), but not among children with high SES (aHR = 0.81 [0.52–1.26], *p* = .345) (Table 2).

Interaction plots based on SES tertiles further confirmed the observed significant interactions. For all asthma‐related outcomes, except for asthma‐like symptoms until age 3, children from low SES families exhibited a stronger positive association between higher WDP scores and increased risk of asthma and wheeze outcomes. In contrast, children from high SES families showed either no association or a trend toward reduced risk with increasing WDP scores. Notably, for asthma‐like symptoms until age 3 years, an opposite pattern was observed: children from high SES families showed a higher predicted risk of asthma‐like symptoms in relation to increasing WDP scores, compared to those from lower SES backgrounds (Figure S2).

In sensitivity analyses, when testing for independent associations of SES components with childhood asthma and wheeze outcomes, we found that the interactions with SES were primarily driven by maternal education level (Figure S3A). There was a significant inverse association between maternal education level and all childhood asthma/wheeze outcomes, except for asthma‐like symptoms up to age 3 years (Table S3). In the mediation analysis, we did not find any significant mediation effect, indicating that the effect of SES and maternal education level on childhood asthma outcomes is not mediated through WDP.

### 3.4 Association between WDP, SES, and childhood asthma/wheeze in VDAART

In the VDAART cohort, univariate analyses revealed that the WDP metabolite‐score during early pregnancy (weeks 10–18), late pregnancy (weeks 32–38), and child age 1 year were all associated with an increased risk of recurrent wheeze until age 3 and asthma until ages 3 and 6 years. After adjusting for prepregnancy maternal BMI, gestational age, smoking during pregnancy, SES, child sex, child weight, child race, and vitamin D intervention, the associations between the child age 1 year WDP metabolite‐score and recurrent wheeze by age 3 remained significant (aOR = 1.48 [95% CI, 1.13–1.95], *p* = .005, FDR‐adjusted *p* = .020) (Table S4). Additionally, significant associations were observed between the WDP metabolite‐score at child 3 years and recurrent wheeze until age 3 and asthma until age 6 years (Table S4).

In VDAART, there were strong negative correlations between SES and WDP during pregnancy and early childhood (Pearson correlation coefficients ranging from −0.45 to −0.20, FDR‐corrected *p*‐values <0.01) (Figure S3B). However, unlike COPSAC_2010_, there was no significant interaction between WDP scores and SES. Yet, SES and its components, that is, maternal education, maternal age, and household income were all identified as independent risk factors for asthma/wheeze outcomes. Higher SES was significantly associated with a reduced risk of wheezing until age 3 (OR = 0.77 [0.69–0.86], *p* < .001) as well as a reduced risk of childhood asthma until age 3 (OR = 0.84 [0.78–0.91], *p* < .001) and age 6 years (OR = 0.85 [0.79–0. 91], *p* < .001) (Table S5, Figure S3B).

### 3.5 Association between WDP, SES, and childhood asthma/wheeze in COPSAC_2000_

In multivariable models adjusted for covariates, the neonatal WDP metabolite‐score was significantly associated with recurrent wheeze up to age 3 (aHR = 1.54 [1.14–2.08], *p* = .005), and asthma up to age 7 years (aHR = 1.45 [1.10–1.90], *p* = .008) (Table S6).

There was a negative correlation between SES and the neonatal DBS WDP metabolite‐score (Pearson correlation coefficient − 0.13, FDR‐corrected *p*‐value = 0.01) (Figure S3C). No significant interaction was observed between the DBS WDP metabolite‐score and SES in relation to asthma/wheeze outcomes. However, alike the COPSAC_2010_ findings, a stratified analysis based on the median split of SES showed significant associations between the DBS WDP metabolite‐score and recurrent wheeze until age 3 (aHR = 1.99 [1.16–3.43], *p* = .013) as well as asthma until age 7 (aHR = 1.85 [1.19–2.87], *p* = .006) among children with low SES, but not in those with high SES (Table S6). We did not find any significant associations between components of socioeconomic status (SES) and childhood asthma outcomes (Table S7).

### 3.6 Subanalysis of the effect of asthma genetic risk on WDP‐asthma associations

To investigate whether the associations between WDP in early childhood and asthma/wheeze outcomes were influenced by the child's genetic risk of asthma, we performed an interaction analysis between the WDP metabolite‐scores and the asthma PRS. In the COPSAC_2010_ cohort, the PRS was available for 533 out of the 594 children included in the study. The analysis showed a significant interaction between the WDP metabolite‐score at 6 months and asthma PRS for number of asthma‐like symptoms until age 3 (*p*‐interaction = 0.005), number of exacerbations until age 6 (*p*‐interaction = 0.018), and asthma up to age 6 (*p*‐interaction = 0.011) and 10 years (*p*‐interaction = 0.004). Analysis stratified by asthma PRS (median split) showed that a higher WDP metabolite score at 6 months was associated with an increased risk of asthma up to age 10 among children in the low PRS group (aHR = 1.89 [1.04–3.46], *p* = .038), whereas in the high PRS group the WDP metabolite score did not show a statistically significant effect (Table S8).

## 4 DISCUSSION

In this study, we examined three mother–child cohorts with longitudinal metabolomics profiles and detailed clinical assessments of asthma/wheeze outcomes to explore the interplay between WDP during pregnancy and early childhood and SES in the development of childhood asthma. In all cohorts, we observed negative correlations between SES and WDP scores, indicating lower SES associated with higher WDP scores. In the COPSAC_2010_ cohort, there was a significant interaction between maternal and child WDP scores and SES in relation to asthma/wheeze outcomes up to age 10, where higher WDP scores were associated with a higher risk of asthma/wheeze outcomes among children with low SES but not among children with high SES. For the number of asthma‐like symptoms up to age 3, children from high‐SES families showed a higher predicted risk. We speculate that this may be due to reporting bias, as parents with higher SES are more likely to complete symptom diaries, particularly when their children are young (i.e., ages 0–3 years). In the VDAART and COPSAC_2000_ replication cohorts, similar associations were observed between higher WDP metabolite scores and an increased risk of asthma/wheeze. Although no significant interaction was observed between WDP scores and SES in the replication cohorts, lower SES was independently associated with an increased risk of asthma/wheeze in VDAART. Further, in COPSAC_2000_, higher neonatal DBS WDP metabolite‐scores were associated with a higher risk of asthma/wheeze among children with low SES but not among those with high SES. These findings support the hypothesis that WDP during pregnancy and early life, combined with lower SES, contributes to the risk of childhood asthma/wheeze, which aligns with a Swedish study involving 955,371 children showing association between lower SES and higher rates of childhood asthma.^33^

In our study, among determinants of SES, we considered household income, maternal education level, and maternal age at child second birthday. Many mothers were at the beginning of their education during pregnancy and continued their education after delivery. To avoid misclassifying this group as low SES, we considered baseline misclassification to pose a greater risk than postexposure misclassification, as educational attainment and income generally follow an upward trajectory and reflect longer‐term socioeconomic positioning.

Notably, we found that the associations of these SES determinants on childhood asthma/wheeze outcomes varied. In the COPSAC_2010_ cohort, maternal education had the strongest association, whereas in the VDAART cohort, all determinants were associated with asthma/wheeze outcomes. These differences may be partly explained by societal differences such as welfare system, healthcare accessibility, and work‐life balance differences between the cohorts from Denmark and the US. The COPSAC_2010_ finding aligns with data obtained from 47,099 children in prospective birth cohort studies across 10 European countries, which showed that children of mothers with lower education levels had a higher prevalence of asthma compared to those whose mothers had higher education levels.^34^ Previous studies have also shown that children from families with lower SES may be more exposed to environmental factors that contribute to asthma, such as indoor pollutants, tobacco smoke, inadequate healthcare, and poor nutrition.^15^

During pregnancy, the maternal diet significantly influences fetal development.^35^ A Westernized diet, as observed in the analysis of FFQ responses in COPSAC_2010_, is characterized by high intakes of saturated fats and processed sugars, which can promote systemic inflammation and contribute to the development and exacerbations of asthma.^10^ Animal models have shown that maternal high‐fat diets (HFDs) during pregnancy may have long‐lasting effects on offspring respiratory health. For instance, MacDonald et al^36^ demonstrated that offspring of mice fed an HFD (60% fat calories) during pregnancy exhibited innate airway hyperresponsiveness, a hallmark of asthma, regardless of their postweaning diet. Furthermore, de Vries and Howie^37^ found that mice on a high‐saturated‐fat diet, when exposed to allergens prior to obesity onset, showed reduced production of proinflammatory cytokines, eotaxin levels, and eosinophilia in the lungs compared to mice on a standard diet. These studies suggest a direct association between diet and asthma pathophysiology including topical airway inflammation and hyperresponsiveness, which aligns with our findings that further suggest a two‐hit hypothesis with low SES worsening the effect of a Westernized diet in pregnancy and early childhood on asthma/wheeze risk.

Our study further demonstrated that a higher WDP score is associated with lower intakes of polyunsaturated fatty acids. Specifically, 3‐carboxy‐4‐methyl‐5‐propyl‐2‐furanpropanoate (CMPF) and hydroxy‐CMPF were the metabolites most strongly and inversely associated with WDP. These metabolites are derivatives of dietary furan fatty acids and serve as markers of fish oil and fatty fish intake.^38^ In the COPSAC_2010_ cohort, we previously showed that CMPF is highly vertically transferred from mothers to newborns^39^ and is associated with a reduced risk of asthma, wheezing, and infections in childhood.^29^, ^40^ Moreover, antioxidant‐related metabolites, such as ergothioneine and stachydrine, and vitamin A derivative metabolites, like beta‐cryptoxanthin and carotene diol, had a negative score in the WDP metabolite‐score. Ergothioneine and stachydrine were also among the metabolites we previously showed can be transferred from mother to child.^39^ These metabolites are related to fish, green vegetables, and fruit intake, and were associated with an overall decreased risk of infections in the COPSAC_2010_ cohort.^39^

Finally, we also observed in our study that the association between the child WDP metabolite‐score at 6 months and asthma appeared stronger in children with low genetic risk of asthma, that is, a low PRS, suggesting that modifiable environmental factors, such as diet, may play a greater role when genetic susceptibility is lower. This observation is exploratory and should be considered hypothesis‐generating. Individuals at lower genetic risk may be more susceptible to external influences that contribute to asthma development. In contrast, among children with high genetic risk, the strong predisposition to asthma may overshadow the associations with diet, implying that genetic factors are the primary drivers of disease risk in this group. However, further studies are needed to validate this finding and to determine whether it is consistent with potential gene–environment interactions.

A key strength of our study is the prospective assessment in the COPSAC_2010_ cohort, which covers the period from pregnancy through childhood. This comprehensive dataset enabled us to include relevant covariates and evaluate phenotypes in relation to childhood asthma/wheeze outcomes over time. Furthermore, we collected detailed information on maternal dietary intake during pregnancy week 20–24 using an FFQ, together with plasma metabolome profiles of the mothers during pregnancy and the children at multiple time points in childhood. Metabolome profiling offers several advantages over FFQs.^41^, ^42^ Unlike FFQs, which rely on self‐reported data and are subject to recall bias, metabolome profiling provides objective biochemical data, enhancing accuracy. It offers a detailed view of dietary intake by detecting a broader range of dietary biomarkers compared to the limited scope of FFQs. Additionally, it more effectively captures environmental interactions, identifies exposures missed by FFQs, and aids in understanding the biological mechanisms underlying diseases. In our study, combining FFQ with untargeted plasma metabolome profiling provides a complementary approach to evaluating WDP. Additionally, to effectively address batch effects in metabolite data and ensure robust normalization as well as ease of comparison of metabolite profiles at different time points in COPSAC_2010_, we leveraged anchor samples—consistent reference samples included in all batches to account for technical variability. This approach enhances data quality and ensures reliability in downstream analyses.

Despite the advantages of our complementary approach in combining FFQ data with longitudinal metabolome profiles, several limitations must be acknowledged. First, the assessment of the WDP in this study for both mothers and children relies on the analysis of FFQ responses collected from mothers in the COPSAC_2010_ cohort. The maternal FFQ score was then used to select the WDP‐related metabolites and calculate WDP metabolite scores for both mothers and children in COPSAC_2010_ as well as in the replication cohorts. This likely influenced the findings, as child scores may not accurately reflect actual child diets, and variations in dietary habits across countries were not accounted for. Secondly, although we adjusted for a broad range of covariates in our regression models, we cannot entirely rule out the potential for confounding effects that may distort the true relationships between WDP, SES, and childhood asthma/wheeze outcomes. For example, maternal history of asthma and other atopic diseases was not included in the analyses of the COPSAC_2010_ cohort. Given the strong heritability of asthma and its potential correlation with maternal dietary behaviors, this may represent a potential source of residual confounding. Moreover, low SES is associated with multiple environmental and psychosocial exposures that were not comprehensively captured in the present study, including housing conditions, air pollution, psychosocial stress, and differences in healthcare access. These factors may independently influence childhood asthma risk and could partially explain the observed associations. Third, diagnostic criteria differed between the COPSAC cohorts and the VDAART cohort. COPSAC outcomes were based on protocol‐driven clinical assessments and symptom diaries, whereas VDAART relied primarily on parental reports of physician‐diagnosed asthma and wheeze. Fourth, due to notable differences between the COPSAC_2010_ cohort and the replication cohorts, we were unable to fully replicate the modifying effect of SES on the associations between WDP scores and asthma‐related outcomes. Specifically, maternal metabolite data were not available in COPSAC_2000_, and metabolomics profiling was conducted at noncomparable time points across cohorts. In addition, both replication cohorts represent high‐risk populations, whereas the discovery cohort is population‐based. These differences precluded pooling of data across cohorts. Therefore, replication in additional population‐based clinical studies is needed to validate our findings.

## 5 CONCLUSION

This study highlights an inverse association between WDP during pregnancy and early childhood and the development of childhood asthma/wheeze phenotypes, suggesting that diet screening and targeted dietary interventions during pregnancy may have potential for risk stratification and prevention efforts. Metabolomics profiling of pregnant mothers to derive a WDP metabolite‐score may provide an objective tool to support dietary guidance, particularly in families with low SES. However, the modifying effect of SES on WDP–asthma associations could not be fully replicated across cohorts, likely due to differences in study design and population characteristics. Future studies in population‐based cohorts are needed to clarify the causal relationship between WDP, SES, and childhood asthma/wheeze outcomes and further explore the underlying biological mechanisms.

## AUTHOR CONTRIBUTIONS

Mina Ali has written the first draft of the manuscript and conducted the analysis. All coauthors provided significant intellectual input and contributed substantially to the interpretation of the analyses. All authors guarantee that the accuracy and integrity of any part of the work have been appropriately investigated and resolved, and all authors have approved the final version of the manuscript.

## FUNDING INFORMATION

COPSAC (Copenhagen Prospective Studies on Asthma in Childhood) is funded by private and public research funds, which are all listed on www.copsac.com. The Ministry of Health (Grant no 903516); Danish Council for Strategic Research (Grant no 0603‐00280B); The Lundbeck Foundation (Grant no R16‐A1694) and The Capital Region Research Foundation have provided core support to the COPSAC research center. This project has received funding from the European Research Council (ERC) under the European Union's Horizon 2020 research and innovation programme (grant agreement No. 946228).

## CONFLICT OF INTEREST STATEMENT

The authors declare no conflicts of interest.

## GOVERNANCE

We are aware of and comply with recognized codes of good research practice, including the Danish Code of Conduct for Research Integrity. We comply with national and international rules on the safety and rights of patients and healthy subjects, including Good Clinical Practice (GCP) as defined in the EU's Directive on Good Clinical Practice, the International Conference on Harmonization's (ICH) good clinical practice guidelines and the Helsinki Declaration. Privacy is important to us, which is why we follow national and international legislation on General Data Protection Regulation (GDPR), the Danish Act on Processing of Personal Data and the practice of the Danish Data Inspectorate.

## Supporting information


**Figure S1** **Characterization of the Western Dietary Pattern (WDP) in the COPSAC**_**2010**_ **Cohort**. This characterization is based on (A) nutrient categories and (B) metabolites. (A) Nutrient categories: The 95 nutrient components were grouped into four categories for ease of visualization: amino acids, fatty acids and sterols, sugars, vitamins, fibers, minerals and inorganics. Polyunsaturated fatty acids were identified as key determinants of Principal Component 2 (PC2). (B) Metabolite associations: Using a sparse partial least squares model, 41 metabolites were selected. Positive metabolite scores indicate a direct association, whereas negative scores suggest an inverse relationship with the Western dietary pattern.
**Figure S2:** Interaction plots displaying the predicted risk of asthma and wheeze outcomes in relation to WDP scores across tertiles of socioeconomic status (SES; Low, Medium, High) in the COPSAC_2010_ cohort. For all plots, there were significant interactions between SES and WDP scores, based on covariate‐adjusted regression analyses.
**Figure S3:** WDP scores, socioeconomic status (SES), and asthma/wheeze outcomes in (A) COPSAC_2010_, (B) VDAART, and (C) COPSAC_2000_ cohorts. Left panels illustrate correlations between SES, its components, and WDP scores. Right panels illustrate associations between SES, its components, and asthma/wheeze outcomes.
**Table S1:** Baseline characteristics of mothers and children in the COPSAC_2010_ cohort for the subgroup who completed the food frequency questionnaire (FFQ) and the subgroup who did not complete the FFQ.
**Table S2:** Association of Western dietary pattern (WDP) scores with childhood asthma and wheeze outcomes in COPSAC_2010_ in multivariate analysis.
**Table S3:** Association of SES and SES determinants with childhood asthma and wheeze outcomes in the COPSAC_2010_ cohort.
**Table S4:** Association of WDP scores with childhood asthma/wheeze outcomes in the VDAART cohort.
**Table S5:** Association of SES and SES determinants with childhood asthma/wheeze outcomes in the VDAART cohort.
**Table S6:** Association of DBS WDP metabolite‐score with childhood asthma and wheeze outcomes in the COPSAC_2000_ cohort, and in SES‐stratified analyses (median split).
**Table S7:** Association of SES and SES determinants with childhood asthma/wheeze outcomes in the COPSAC_2000_ cohort.
**Table S8:** Association of WDP scores with childhood asthma/wheeze outcomes in low and high PRS groups in the COPSAC_2010_ cohort in multivariate analysis.
**Table S9:** Asthma and wheeze outcomes used in the discovery and replication cohorts.

## Data Availability

The first and last authors had full access to the data and held final responsibility for the decision to submit the manuscript for publication.
