# Supplementary material for: Western dietary pattern during pregnancy and early childhood increases risk of childhood asthma dependent on socioeconomic status
Source: Pediatr Allergy Immunol. 2026 Apr 17;37(4):e70351. doi: 10.1111/pai.70351 (PMC13090432; doi:10.1111/pai.70351)
Supplement: Supplementary file 1 — Figure S1 Characterization of the Western Dietary Pattern (WDP) in the COPSAC 2010 Cohort. This characterization is based on (A) nutrient categories and (B) metabolites. (A) Nutrient categories: The 95 nutrient components were grouped into four categories for ease of visualization: amino acids, fatty acids and sterols, sugars, vitamins, fibers, minerals and inorganics. Polyunsaturated fatty acids were identified as key determinants of Principal Component 2 (PC2). (B) Metabolite associations: Using a sparse partial least squares model, 41 metabolites were selected. Positive metabolite scores indicate a direct association, whereas negative scores suggest an inverse relationship with the Western dietary pattern. Figure S2: Interaction plots displaying the predicted risk of asthma and wheeze outcomes in relation to WDP scores across tertiles of socioeconomic status (SES; Low, Medium, High) in the COPSAC2010 cohort. For all plots, there were significant interactions between SES and WDP scores, based on covariate‐adjusted regression analyses. Figure S3: WDP scores, socioeconomic status (SES), and asthma/wheeze outcomes in (A) COPSAC2010, (B) VDAART, and (C) COPSAC2000 cohorts. Left panels illustrate correlations between SES, its components, and WDP scores. Right panels illustrate associations between SES, its components, and asthma/wheeze outcomes. Table S1: Baseline characteristics of mothers and children in the COPSAC2010 cohort for the subgroup who completed the food frequency questionnaire (FFQ) and the subgroup who did not complete the FFQ. Table S2: Association of Western dietary pattern (WDP) scores with childhood asthma and wheeze outcomes in COPSAC2010 in multivariate analysis. Table S3: Association of SES and SES determinants with childhood asthma and wheeze outcomes in the COPSAC2010 cohort. Table S4: Association of WDP scores with childhood asthma/wheeze outcomes in the VDAART cohort. Table S5: Association of SES and SES determinants with childhood asthma/whee [file PAI-37-e70351-s001.docx]

# **Supplementary figures**

**Supplementary figure 1**

1. **Westernized dietary pattern based on nutrient categories**

1. **Westernized dietary pattern based on metabolites**

**Supplementary Figure 1:** **Characterization of the Western Dietary Pattern (WDP) in the COPSAC_2010_ Cohort.** This characterization is based on (A) nutrient categories and (B) metabolites. **(A)** Nutrient categories: The 95 nutrient components were grouped into four categories for ease of visualization: amino acids, fatty acids and sterols, sugars, vitamins, fibers, minerals and inorganics. Polyunsaturated fatty acids were identified as key determinants of Principal Component 2 (PC2). **(B)** Metabolite associations: Using a sparse partial least squares model, 41 metabolites were selected. Positive metabolite scores indicate a direct association, whereas negative scores suggest an inverse relationship with the Western dietary pattern.

**Supplementary figure 2**

**Supplementary Figure 2:** **Interaction plots displaying the predicted risk of asthma and wheeze outcomes in relation to WDP scores across tertiles of socioeconomic status (SES; Low, Medium, High) in the COPSAC_2010_ cohort.** For all plots, there were significant interactions between SES and WDP scores, based on covariate-adjusted regression analyses.

**Supplementary figure 3**

**A)**

**B)**

**C)**

**Supplementary Figure 3:** WDP scores, socioeconomic status (SES), and asthma/wheeze outcomes in (A) COPSAC_2010_, (B) VDAART, and (C) COPSAC_2000_ cohorts. Left panels illustrate correlations between SES, its components, and WDP scores. Right panels illustrate associations between SES, its components, and asthma/wheeze outcomes.

# **Supplementary tables**

**Supplementary Table 1:** Baseline characteristics of mothers and children in the COPSAC_2010_ cohort for the subgroup who completed the food frequency questionnaire (FFQ) and the subgroup who did not complete the FFQ.

| Baseline characteristics | Included | Not included | P-value |
| --- | --- | --- | --- |
| Mothers |  |  |  |
| Number | 594 | 106 |  |
| Age at birth (years) (mean (SD)) | 32.23 (4.3) | 32.47 (4.69) | 0.635 |
| Gestational age at birth (days) (mean (SD)) | 279.21 (11.29) | 278.09 (13.49) | 0.425 |
| Maternal pre-pregnancy Body Mass Index (mean (SD)) | 24.53 (4.35) | 24.62 (4.64) | 0.852 |
| Smoking during pregnancy (Yes/No) | 48/546 | 6/100 | 0.507 |
| Alcohol during pregnancy (Yes/No) | 86/507 | 15/90 | 1 |
| Maternal education at birth  Low/Medium/High | 43/380/171 | 8/64/34 | 0.768 |
| Annual income  Low/Medium/High | 52/319/223 | 8/57/41 | 0.912 |
| High dose vitamin D in the third trimester of pregnancy (Yes/No) | 237/245 | 58/41 | 0.110 |
| High dose PUFA in the third trimester of pregnancy (Yes/No) | 290/302 | 56/47 | 0.367 |
| Children |  |  |  |
| Number | 594 | 106 |  |
| Gender, Female/ Male | 286/308 | 54/52 | 0.671 |
| Birth weight (Kilograms)  (mean (SD)) | 3.56(0.54) | 3.45(0.56) | 0.06 |
| Birth season Winter/Spring/Summer/Fall | 143/177/139/133 | 69/9/10/15 | **< 0.05** |
| Cesarean delivery (Yes/No) | 130/464 | 21/85 | 0.726 |
| Solely breast-fed days (Days)  (mean (SD)) | 104.36(59.32) | 96.62(61.41) | 0.235 |
| Asthma ever at age 10 (Yes/No) | 154/440 | 28/78 | 0.905 |

P-values summarize the characteristic differences between the two cohorts. Low, medium, and high-level educational attainment are defined as "primary, secondary, or college graduate," "tradesman or bachelor's degree," and "master’s degree or higher," respectively. Low, medium, and high annual income are defined as <50,000, 50,000 - 110,000, and >110,000 euros, respectively.

**Supplementary Table 2:** Association of Western dietary pattern (WDP) scores with childhood asthma and wheeze outcomes in COPSAC_2010_ in multivariate analysis.

|  | Analysis | FFQ-score pregnancy (week 24-28) | Metabolite-score pregnancy (week 24) | Metabolite-score child 6 months | Metabolite-score child 18 months |
| --- | --- | --- | --- | --- | --- |
| Asthma-like symptoms at age 0-3 years | Quasi-Poisson | 1.03 [0.99-1.07] (P = 0.141)  (n=552) | 1.07 [1.02-1.12] (P = 0.007, q =0.028)  (n=544) | 1.04 [0.97-1.12] (P = 0.286)  (n=474) | 0.97 [0.89-1.05] (P = 0.468)  (n=489) |
| Recurrent wheeze at age 0-3 years | Cox | 1.11 [0.94-1.31] (P = 0.225)  (n=576) | 1.00 [0.84-1.20] (P = 0.976)  (n=568) | 1.01 [0.76-1.33] (P = 0.956)  (n=493) | 1.31 [0.95-1.81] (P = 0.105)  (n=509) |
| Number of exacerbations at age 0-6 years | Quasi-Poisson | 0.99 [0.79-1.23] (P = 0.899)  (n=576) | 0.95 [0.75-1.22] (P = 0.704)  (n=568) | 1.12 [0.78-1.64] (P = 0.538)  (n=493) | 1.05 [0.68-1.64] (P = 0.817)  (n=509) |
| Asthma at age 0-6 years | Cox | 1.04 [0.86-1.25] (P = 0.697)  (n=576) | 0.98 [0.80-1.19] (P = 0.814)  (n=568) | 1.29 [0.94-1.78] (P = 0.119)  (n=493) | 1.02 [0.71-1.47] (P = 0.904)  (n=509) |
| Asthma at age 0-10 years | Cox | 1.06 [0.89-1.26] (P = 0.502)  (n=576) | 1.01 [0.84-1.21] (P = 0.946)  (n=568) | 1.22 [0.91-1.64] (P = 0.179)  (n=493) | 1.02 [0.73-1.42] (P = 0.912)  (n=509) |

The analysis is adjusted for pre-pregnancy maternal BMI, gestational age, cesarean delivery, maternal smoking and alcohol consumption during pregnancy, child sex, birth weight, birth season, length of solely breastfeeding, daycare start age, socioeconomic status (PCA based on household income, maternal education level, and maternal age at child’s second birthday), and n-3 LCPUFA and vitamin D interventions. **q** denotes the Benjamini-Hochberg adjusted P value.

**Supplementary Table 3:** Association of SES and SES determinants with childhood asthma and wheeze outcomes in the COPSAC_2010_ cohort.

|  | Analysis | SES | Maternal education | Maternal age | Household income |
| --- | --- | --- | --- | --- | --- |
| Asthma-like symptoms at age 0-3 years | Quasi-Poisson | 1.01 [0.98-1.05]  (P = 0.449)  (n=564) | 1.05 [0.98-1.12]  (P = 0.152)  (n=564) | 1.00 [0.99-1.01] (P = 0.801)  (n=564) | 1.00 [0.97-1.03] (P = 0.988)  (n=564) |
| Recurrent wheeze at age 0-3 years | Cox | 0.90 [0.77-1.06] (P = 0.205)  (n=594) | 0.68 [0.51-0.89] (P = 0.006)  (n=594) | 0.98 [0.95-1.02] (P = 0.315)  (n=594) | 1.05 [0.92-1.20] (P = 0.435)  (n=594) |
| Number of exacerbations at age 0-6 years | Quasi-Poisson | 1.05 [0.85-1.31] (P = 0.630)  (n=594) | 0.73 [0.58-0.90] (P = 0.004)  (n=594) | 1.02 [0.97-1.08] (P = 0.338)  (n=594) | 1.17 [0.98-1.41] (P = 0.084)  (n=594) |
| Asthma at age 0-6 years | Cox | 0.91 [0.76-1.08] (P = 0.286)  (n=594) | 0.62 [0.45-0.85] (P = 0.003)  (n=594) | 0.99 [0.95-1.03] (P = 0.615)  (n=594) | 1.07 [0.93-1.25] (P = 0.341)  (n=594) |
| Asthma at age 0-10 years | Cox | 0.97 [0.82-1.13] (P = 0.665)  (n=594) | 0.76 [0.57-1.00] (P = 0.053)  (n=594) | 1.00 [0.97-1.04] (P = 0.795)  (n=594) | 1.05 [0.92-1.20] (P = 0.505)  (n=594) |

**Supplementary Table 4:** Association of WDP scores with childhood asthma/wheeze outcomes in the VDAART cohort.

|  | Analysis | Metabolite-score pregnancy (week 10-18) | Metabolite-score pregnancy (week 32-38) | Metabolite-score child 1 year | Metabolite-score child 3 years |
| --- | --- | --- | --- | --- | --- |
| Recurrent wheeze at age 0-3 years | Logistic regression | 1.28 [1.09-1.51]  (P = 0.004) (n=719) | 1.29 [1.09-1.53] (P = 0.003) (n=719) | 1.60 [1.31-1.98] (P < 0.001) (n=469) | 1.41 [1.12-1.78] (P = 0.004) (n=411) |
|  |  | 1.03 [0.78-1.37] (P = 0.809) (n=375) | 1.13 [0.86-1.49] (P = 0.376) (n=375) | 1.48 [1.13-1.95] (P = 0.005) (n=375) | 1.16 [0.81-1.67] (P = 0.430) (n=184) |
| Asthma at age 0-3 years | Cox | 1.18 [1.05-1.31] (P = 0.003) (n=775) | 1.12 [1.00-1.25] (P = 0.050) (n=773) | 1.23 [1.07-1.41] (P = 0.004) (n=469) | 1.14 [0.98-1.32] (P = 0.101) (n=411) |
|  |  | 1.08 [0.89-1.30] (P = 0.450) (n=375) | 1.06 [0.88-1.28] (P = 0.548) (n=375) | 1.16 [0.96-1.40] (P = 0.121) (n=375) | 1.24 [0.96-1.59] (P = 0.100) (n=184) |
| Asthma at age 0-6 years | Cox | 1.16 [1.04-1.29] (P = 0.005) (n=775) | 1.12 [1.01-1.25] (P = 0.034) (n=773) | 1.22 [1.07-1.39] (P = 0.004) (n=469) | 1.15 [1.00-1.34] (P = 0.054) (n=411) |
|  |  | 1.03 [0.86-1.23] (P = 0.755) (n=375) | 1.04 [0.87-1.24] (P = 0.682) (n=375) | 1.16 [0.97-1.39] (P = 0.103) (n=375) | 1.23 [0.96-1.56] (P = 0.096) (n=184) |

First-row associations are related to univariate analysis, and second-row associations are related to multivariate analysis where we adjusted for pre-pregnancy maternal BMI, gestational age, smoking during pregnancy, SES, child sex, child weight, child race and vitamin D intervention. All significant associations (P value < 0.05) remained significant after Benjamini-Hochberg FDR correction.

**Supplementary Table 5:** Association of SES and SES determinants with childhood asthma/wheeze outcomes in the VDAART cohort.

|  | Analysis | SES | Maternal education | Maternal age | Household income |
| --- | --- | --- | --- | --- | --- |
| Recurrent wheeze at age 0-3 years | Logistic regression | 0.77 [0.69-0.86]  (P < 0.001) (n=748) | 0.79 [0.68-0.92]  (P = 0.002) (n=718) | 0.94 [0.91-0.97] (P < 0.001) (n=718) | 0.77 [0.67-0.88] (P < 0.001) (n=546) |
| Asthma at age 0-3 years | Cox | 0.84 [0.78-0.91] (P < 0.001) (n=806) | 0.86 [0.77-0.95] (P = 0.004) (n=772) | 0.95 [0.93-0.97] (P < 0.001) (n=772) | 0.87 [0.80-0.94] (P = 0.001) (n=585) |
| Asthma at age 0-6 years | Cox | 0.85 [0.79–0.91] (P < 0.001) (n=806) | 0.86 [0.78-0.95] (P = 0.003) (n=772) | 0.95 [0.93-0.97] (P < 0.001) (n=772) | 0.88 [0.81-0.95] (P = 0.002) (n=585) |

**Supplementary Table 6:** Association of DBS WDP metabolite-score with childhood asthma and wheeze outcomes in the COPSAC_2000_ cohort, and in SES-stratified analyses (median split).

|  | Analysis | Metabolite-score child 1-12 days,  Entire cohort | Metabolite-score child 1-12 days,  Low SES | Metabolite-score child 1-12 days,  High SES |
| --- | --- | --- | --- | --- |
| Asthma-like symptoms at age 0-3 years | Quasi-Poisson | 1.02 [0.89-1.17]  (P = 0.810)  n=246 | 0.95 [0.78-1.16]  (P = 0.629)  n=110 | 0.99 [0.82-1.21]  (P = 0.926)  n=136 |
| Recurrent wheeze at age 0-3 years | Cox | 1.54 [1.14-2.08] (P = 0.005)  n=293 | 1.99 [1.16-3.43]  (P = 0.013)  n=138 | 1.38 [0.89-2.16]  (P = 0.150)  n=155 |
| Number of exacerbations at age 0-6 years | Quasi-Poisson | 2.76 [0.81-9.44] (P = 0.104)  n=268 | 1.82 [0.46-7.17]  (P = 0.390)  n=125 | 0.73 [0.18-2.92]  (P = 0.657)  n=143 |
| Asthma at age 0-7 years | Cox | 1.45 [1.10-1.90] (P = 0.008)  n=293 | 1.85 [1.19-2.87]  (P = 0.006)  n=138 | 1.21 [0.80-1.83]  (P = 0.358)  n=155 |
| Asthma at age 0-12 years | Logistic | 0.90 [0.62-1.28] (P = 0.560)  n=279 | 0.90 [0.51-1.54]  (P = 0.692)  n=129 | 0.76 [0.45-1.27]  (P = 0.304)  n=150 |

The analyses are adjusted for gestational age, cesarean delivery, smoking during pregnancy, alcohol consumption during pregnancy, child sex, birth weight, birth season, duration of exclusive breastfeeding, daycare start age, and SES (in the whole cohort).

**Supplementary Table 7:** Association of SES and SES determinants with childhood asthma/wheeze outcomes in the COPSAC_2000_ cohort.

|  | Analysis | SES | Maternal education | Maternal age | Household income |
| --- | --- | --- | --- | --- | --- |
| asthma-like symptoms at age 0-3 years | Quasi-Poisson | 1.03 [0.91-1.16] (P = 0.681)  n=270 | 0.96 [0.81-1.13] (P = 0.628)  n=270 | 1.02 [0.99-1.04] (P = 0.226)  n=270 | 1.00 [0.89-1.13] (P = 0.949)  n=270 |
| recurrent wheeze at age 0-3 years | Cox | 1.13 [0.89-1.44] (P = 0.329)  n=359 | 0.97 [0.69-1.37] (P = 0.882)  n=359 | 1.01 [0.96-1.07] (P = 0.729)  n=359 | 1.24 [0.99-1.56] (P = 0.064)  n=359 |
| number of exacerbations at age 0-6 years | Quasi-Poisson | 0.76 [0.29-1.74]  (P = 0.548)  n=299 | 0.58 [0.11-1.75] (P = 0.406)  n=299 | 0.93 [0.77-1.11] (P = 0.452)  n=299 | 1.12 [0.45-2.37] (P = 0.780)  n=299 |
| asthma at age 0-7 years | Cox | 1.10 [0.87-1.39]  (P = 0.427)  n=359 | 0.91 [0.65-1.26] (P = 0.567)  n=359 | 1.02 [0.97-1.07] (P = 0.452)  n=359 | 1.17 [0.94-1.46] (P = 0.150)  n=359 |
| asthma at age 0-12 years | Logistic | 1.13 [0.85-1.48] (P = 0.398)  n=325 | 0.98 [0.66-1.41] (P = 0.899)  n=325 | 1.02 [0.96-1.09] (P = 0.525)  n=325 | 1.19 [0.90-1.55] (P = 0.211)  n=325 |

**Supplementary Table 8:** Association of WDP scores with childhood asthma/wheeze outcomes in low and high PRS groups in the COPSAC_2010_ cohort in multivariate analysis.

|  | Analysis | PRS | Metabolite-score child 6 months | Interaction P-value | | Metabolite-score child 18 months | Interaction P-value |
| --- | --- | --- | --- | --- | --- | --- | --- |
| Asthma-like symptoms at age 0-3 years | Quasi-Poisson | Low | 1.02 [0.91-1.15] (P = 0.686)  (n=212) | 0.005 | | 1.04 [0.92-1.18] (P = 0.524)  (n=215) | 0.979 |
|  |  | High | 1.00 [0.90-1.10] (P = 0.961)  (n=225) |  |  | 0.93 [0.82-1.06] (P = 0.288)  (n=228) |  |
| Recurrent wheeze at age 0-3 years | Cox | Low | 0.91 [0.56-1.47] (P = 0.704)  (n=225) | 0.607 | | 1.15 [0.69-1.92] (P = 0.599)  (n=227) | 0.578 |
|  |  | High | 1.10 [0.74-1.64] (P = 0.636)  (n=231) |  |  | 1.76 [1.08-2.85] (P = 0.022)  (n=235) |  |
| Number of exacerbations at age 0-6 years | Quasi-Poisson | Low | 1.97 [0.98-4.09] (P = 0.063)  (n=225) | 0.018 | | 0.70 [0.30-1.61] (P = 0.409)  (n=227) | 0.477 |
|  |  | High | 0.95 [0.57-1.58] (P = 0.856)  (n=231) |  |  | 1.55 [0.84-2.90] (P = 0.166)  (n=235) |  |
| Asthma at age 0-6 years | Cox | Low | 1.95 [1.00-3.78] (P = 0.049)  (n=225) | 0.011 | | 0.87 [0.47-1.62] (P = 0.664)  (n=227) | 0.889 |
|  |  | High | 1.13 [0.73-1.76] (P = 0.577)  (n=231) |  |  | 1.29 [0.77-2.17] (P = 0.334)  (n=235) |  |
| Asthma at age 0-10 years | Cox | Low | 1.89 [1.04-3.46] (P = 0.038)  (n=225) | 0.004 | | 0.81 [0.46-1.40] (P = 0.446)  (n=227) | 0.535 |
|  |  | High | 1.09 [0.73-1.63] (P = 0.661)  (n=231) |  | 1.34 [0.84-2.14]  (P = 0.222)  (n=235) | |  |

The analyses are adjusted for pre-pregnancy maternal BMI, gestational age, cesarean delivery, maternal smoking and alcohol consumption during pregnancy, child sex, birth weight, birth season, length of solely breastfeeding, daycare start age, SES, n-3 LCPUFA and vitamin D interventions. Interaction P-values correspond to the P-values for the interaction between WDP scores and PRS in multivariate analysis.

**Supplementary Table 9:** Asthma and wheeze outcomes used in the discovery and replication cohorts.

| Asthma Outcomes | COPSAC_2010_ | COPSAC_2000_ | VDAART |
| --- | --- | --- | --- |
| Asthma-like symptoms at age 0-3 years | X | X |  |
| Recurrent wheeze at age 0-3 years | X | X | X |
| Number of exacerbations at age 0-6 years | X | X |  |
| Asthma at age 0-3 years |  |  | X |
| Asthma at age 0-6 years | X |  | X |
| Asthma at age 0-10 years | X |  |  |
| Asthma at age 0-7 years |  | X |  |
| Asthma at age 0-12 years |  | X |  |
